# Supplementary material for: Proposal for an objective binary benchmarking framework that validates each other for comparing MCDM methods through data analytics
Source: PeerJ Comput Sci. 2023 Apr 25;9:e1350. doi: 10.7717/peerj-cs.1350 (PMC10159627; doi:10.7717/peerj-cs.1350)
Supplement: Supplemental Information 4 [file peerj-cs-09-1350-s004.docx]

**Appendix**

Appendix 1: Calculation Steps and Formulas of MCDM Methods and SWARA and CRITIC Method Applied in This Study.

| ***Stage.*** | ***MABAC*** | ***TOPSIS*** | ***ELECTRE III*** |
| --- | --- | --- | --- |
| **1** | By applying Min-Max normalization, the first decision matrix is normalized with rows and n columns.  $F_{ij}=\frac{f_{ij}-{min}_{i\in m}f_{ij}}{{max}_{i\in m}f_{ij}-{min}_{i\in m}f_{ij}} i\in\left\{ 1,2,\ldots,m \right\}; j\in\left\{ 1,2,\ldots,n \right\} for benefit objectives$  $F_{ij}=\frac{{max}_{i\in m}f_{ij}-f_{ij}}{{max}_{i\in m}f_{ij}-{min}_{i\in m}f_{ij}} i\in\left\{ 1,2,\ldots,m \right\}; j\in\left\{ 1,2,\ldots,n \right\} for cost objectives$ | Application of vector normalization to the first decision matrix.  $F_{ij}=\frac{f_{ij}}{\sqrt{\sum_{i=1}^{m} f_{ij}^{2}}}$ | Conversion of the objective matrix as in ELECTRE II. |
| **2** | After normalization, a weighted decision matrix is created with the following equation.  $v_{ij}=(1+F_{ij})\times w_{j} i\in\left\{ 1,2,\ldots,m \right\}; j\in\left\{ 1,2,\ldots,n \right\}$ | weighted normalized matrix    $v_{ij}=F_{ij}\times w_{j}$ | An element of the concordance matrix of m rows and columns is computed by  $C\left( a,b \right)=\sum_{j=1}^{n} w_{j}C_{j}\left( a,b \right)$  where $C_{j}\left( a,b \right)=$  $\left\{ \begin{matrix} 1 & if F_{j}\left( b \right)-F_{j}\left( a \right)\leq Q_{j} \\ 0 & if F_{j}\left( b \right)-F_{j}\left( a \right)>P_{j} \\ \frac{P_{j}-[F_{j}\left( b \right)-F_{j}\left( a \right)]}{P_{j}-Q_{j}} & if{Q_{j}<F}_{j}\left( b \right)-F_{j}\left( a \right)\leq P_{j} \end{matrix} \right\}$ |
| **3** | The approximate border area for each target is determined.  $b_{j}=\left( \prod_{i=1}^{m} v_{ij} \right)^{\frac{1}{m}}$  $j\in\left\{ 1,2,\ldots,n \right\}$ | Positive and negative ideal solutions  $A^{+}=\left\{ \left( {Max}_{i}\left( v_{ij} \right)│j\in J \right),\left( {Min}_{i}\left( v_{ij} \right)│j\in J^{'} \right)│i\in1, 2, \ldots, m \right\}=\left\{ v_{1}^{+},v_{2}^{+},v_{3}^{+},\ldots,v_{j}^{+}, \ldots, v_{n}^{+} \right\}$  $A^{-}=\left\{ \left( {Min}_{i}\left( v_{ij} \right)│j\in J \right),\left( {Max}_{i}\left( v_{ij} \right)│j\in J^{'} \right)│i\in1, 2, \ldots, m \right\}=\left\{ v_{1}^{-},v_{2}^{-},v_{3}^{-},\ldots,v_{j}^{-}, \ldots, v_{n}^{-} \right\}$ | Construct elements of discordance matrix by  $D_{j}\left( a,b \right)=$  $\left\{ \begin{matrix} 1 & if F_{j}\left( b \right)-F_{j}\left( a \right)>V_{j} \\ 0 & if F_{j}\left( b \right)-F_{j}\left( a \right)\leq P_{j} \\ \frac{F_{j}\left( b \right)-F_{j}\left( a \right)-P_{j}}{V_{j}-P_{j}} & if{P_{j}<F}_{j}\left( b \right)-F_{j}\left( a \right)\leq V_{j} \end{matrix} \right\}$ |
| **4** | The evaluation score of each non-dominant solution is calculated.  $Q_{i}=\sum_{j=1}^{n} (v_{ij}-b_{j})$  $i\in\left\{ 1,2,\ldots,m \right\}$ | The positive (PIS) and negative ideals’ distance values (NIS)  $S_{i+}=\sqrt{\sum_{j=1}^{n} \left( v_{ij}-v_{j}^{+} \right)^{2}} i=1, 2, 3, \ldots, m$  $S_{i-}=\sqrt{\sum_{j=1}^{n} \left( v_{ij}-v_{j}^{-} \right)^{2}} i=1, 2, 3, \ldots, m$ | The credibility matrix of m rows and columns is given by  $S\left( a,b \right)=\left\{ \begin{aligned} C\left( a,b \right) if D_{j}\left( a,b \right)\leq C\left( a,b \right)\forall j \\ C\left( a,b \right)\prod_{j\in J(a,b)} \frac{1-D_{j}\left( a,b \right)}{1-C\left( a,b \right)} otherwise \end{aligned} \right.$ |
| **5** | The optimal solution having the largest Q_i is the recommended solution | Computing relative proximity to ideal solution  $C_{i}=\frac{S_{i-}}{S_{i-}+S_{i+}}$ | Make the selection based on the credibility matrix by calculating the difference between the strength (sum of row) and weakness (sum of column) of each solution, with or without cut-off values. |

| **Stage** | ***CODAS*** | ***SWARA*** | ***FUCA*** |
| --- | --- | --- | --- |
| **1** | The first decision matrix is Max. normalized using the normalization technique.  $F_{ij}=\frac{f_{ij}}{{max}_{i\in m}f_{ij}} i\in\left\{ 1,2,\ldots,m \right\}; j\in\left\{ 1,2,\ldots,n \right\} for benefit$  $F_{ij}=\frac{{min}_{i\in m}f_{ij}}{f_{ij}} i\in\left\{ 1,2,\ldots,m \right\}; j\in\left\{ 1,2,\ldots,n \right\} for cost$ | The criteria are ranked from important to insignificant according to the level of importance decided by the decision maker. The first criterion is the most important and the last criterion is the least important. Next, the decision maker needs to give the relative importance *Sj* value (≥ 0 and ≤ 1), which is the objective *j* over the previous objective (j-1), starting from the second objective (i.e., j = 2, 3, …, n). | For each criterion, rank 1 is assigned to the best value, and rank m is assigned to the worst value. |
| **2** | Construct weighted normalized objective matrix  $v_{ij}=F_{ij}\times w_{j} i\in\left\{ 1,2,\ldots,m \right\};$  $j\in\left\{ 1,2,\ldots,n \right\}$  And determine the negative-ideal solution, $A^{-}$  $A^{-}=\{({Min}_{i}(v_{ij})\vert i \in1, 2, \ldots, m\}$  $=\{v_{1}^{-}, v_{2}^{-}, v_{3}^{-}, \ldots, v_{j}^{-}, \ldots, v_{n}^{-}\}$ | Compute K_j_ and Q_j_  $K_{j}=\left\{ \begin{aligned} 1 ifj=1 \\ S_{j}+1 if j>1 \end{aligned} \right.$  $Q_{j}=\left\{ \begin{aligned} 1 ifj=1 \\ \frac{Q_{j-1}}{K_{j}} if j>1 \end{aligned} \right.$ | A weighted summation for each optimal solution i is computed  $v_{i}=\sum_{j=1}^{n} {(r}_{ij}\times w_{j})$ |
| **3** | Calculate the Euclidean and Taxicab distances  $E_{i}=\sqrt{\sum_{j=1}^{n} \left( v_{ij}-v_{j}^{-} \right)^{2}} i=1,2,3,\ldots,m$  $T_{i}=\sum_{j=1}^{n} \vert v_{ij}-v_{j}^{-}\vert i=1,2,3,...,m$ | Ultimately the weight for each objective is determined as  $w_{j}=\frac{Q_{j}}{\sum_{k=1}^{n} Q_{j}}$  $j\in\left\{ 1,2,\ldots,n \right\}$ |  |
| **4** | Construct the relative assessment matrix  $h_{ik}=\left( E_{i}-E_{k} \right)+\psi\left( E_{i}-E_{k} \right)$  $\times\left( T_{i}-T_{k} \right) i,k\in\left\{ 1,2,\ldots,m \right\}$  And calculate the assessment score of each solution  $H_{i}=\sum_{k=1}^{m} h_{ik} i=1,2,3,...,m$ |  |  |

| **St.** | **COPRAS** | **VIKOR** |
| --- | --- | --- |
| **1** | Construct normalized objective matrix  $F_{ij}=\frac{f_{ij}}{\sum_{k=1}^{m} f_{kj}}$  $i\in\left\{ 1,2,\ldots,m \right\}; j\in\{1,2,\ldots,n\}$ | For each criterion, determine the best and worst values  $F_{j}^{+}= {Max}_{i\in m}f_{ij}$and $F_{j}^{-}= {Min}_{i\in m}f_{ij}$for maximization objectives  $F_{j}^{+}= {Min}_{i\in m}f_{ij}$and $F_{j}^{-}= {Max}_{i\in m}f_{ij}$for minimization objectives |
| **2** | Construct weighted normalized objective matrix  $v_{ij}=F_{ij}\times w_{j}$  $i\in\left\{ 1,2,\ldots,m \right\}; j\in\left\{ 1,2,\ldots,n \right\}$ | Compute *S_i_* and *R_i_*  for each solution  $S_{i}=\sum_{j=1}^{n} w_{j}\left( \frac{F_{j}^{+}-f_{ij}}{F_{j}^{+}-F_{j}^{-}} \right)$  $R_{i}={Max}_{j\in n}\left[ w_{j}\left( \frac{F_{j}^{+}-f_{ij}}{F_{j}^{+}-F_{j}^{-}} \right) \right]$ |
| **3** | For each solution, calculate the sums of weighted normalized values for both benefit and cost objectives  $S_{i+}= \sum_{j=1}^{g} v_{ij}$  $i\in\left\{ 1,2,\ldots,m \right\}$  $S_{i-}= \sum_{j=g+1}^{n} v_{ij}$  $i\in\left\{ 1,2,\ldots,m \right\}$ | Compute $Q_{i}$  $Q_{i}=$  $\gamma\left( \frac{S_{i}-S^{+}}{S^{-}-S^{+}} \right)+$  $(1-\gamma)\left( \frac{R_{i}-R^{+}}{R^{-}-R^{+}} \right)$  $\mathrm{where}{S^{+}= Min}_{i\in m}S_{i,}$ ${S^{-}= Max}_{i\in m}S_{i}$, ${R^{+}= Min}_{i\in m}R_{i}$, ${R^{-}= Max}_{i\in m}R_{i}$ |
| **4** | Determine the relative importance of each solution  $Q_{i}=\left\{ \begin{aligned} S_{i+}+\frac{\sum_{i=1}^{m} S_{i-}}{S_{i-}\sum_{i=1}^{m} \frac{1}{S_{i-}}} for both benefit &and cost \\ S_{i+} for only benefit \\ \frac{\sum_{i=1}^{m} S_{i-}}{S_{i-}\sum_{i=1}^{m} \frac{1}{S_{i-}}} for only cost \end{aligned} \right.$ | Rank Pareto-optimal solutions, sorting by the value of $Q_{i}\vert i=1,2, \ldots,m$ in decreasing order. Propose a compromise solution A^(1)^ by the measure ${Min}_{i\in m}Q_{i}$  if acceptable advantage and acceptable stability in decision-making is met. |

| ***Stage.*** | ***CRITIC*** | ***CoCoSo*** | ***CRADIS*** |
| --- | --- | --- | --- |
| **1** | Min-max normalization type is applied to the overall decision matrix. With M rows and n columns;  $F_{ij}=\frac{f_{ij}-{min}_{i\in m}f_{ij}}{{max}_{i\in m}f_{ij}-{min}_{i\in m}f_{ij}} i\in\left\{ 1,2,\ldots,m \right\}; j\in\left\{ 1,2,\ldots,n \right\} for benefit objectives$  $F_{ij}=\frac{{max}_{i\in m}f_{ij}-f_{ij}}{{max}_{i\in m}f_{ij}-{min}_{i\in m}f_{ij}} i\in\left\{ 1,2,\ldots,m \right\}; j\in\left\{ 1,2,\ldots,n \right\} for cost objectives$ | Min-max normalization type is applied to the overall decision matrix. With M rows and n columns;  $F_{ij}=\frac{f_{ij}-{min}_{i\in m}f_{ij}}{{max}_{i\in m}f_{ij}-{min}_{i\in m}f_{ij}} i\in\left\{ 1,2,\ldots,m \right\}; j\in\left\{ 1,2,\ldots,n \right\} for benefit objectives$  $F_{ij}=\frac{{max}_{i\in m}f_{ij}-f_{ij}}{{max}_{i\in m}f_{ij}-{min}_{i\in m}f_{ij}} i\in\left\{ 1,2,\ldots,m \right\}; j\in\left\{ 1,2,\ldots,n \right\} for cost objectives$ | The first decision matrix is Max. normalized using the normalization technique.  $F_{ij}=\frac{f_{ij}}{{max}_{i\in m}f_{ij}} i\in\left\{ 1,2,\ldots,m \right\}; j\in\left\{ 1,2,\ldots,n \right\} for benefit$  $F_{ij}=\frac{{min}_{i\in m}f_{ij}}{f_{ij}} i\in\left\{ 1,2,\ldots,m \right\}; j\in\left\{ 1,2,\ldots,n \right\} for cost$ |
| **2** | By measuring the dependence (e.g., correlation) between any two criteria, the binary correlation matrix is created.  $\rho_{jk}=\frac{\sum_{i=1}^{m} (F_{ij}-\overline{F_{j}})(F_{ik}-\overline{F_{k}})}{\sqrt{{\sum_{i=1}^{m} (F_{ij}-\overline{F_{j}})}^{2}}\sqrt{{\sum_{i=1}^{m} (F_{ik}-\overline{F_{k}})}^{2}}} j,k\in\left\{ 1,2,\ldots,n \right\}$ | Obtain some of the power weight of the comparability array for each alternative, Si and Pi, respectively: $S_{i}=\sum_{j=1}^{n} (w_{j}r_{ij}).$  This value achieved based on grey relational generation approach | The weighted decision matrix is obtained by the following equation.  $s_{ij}=u_{ij}.w_{jIN}$ |
| **3** | First, the standard deviation of the criteria is calculated.  $\sigma_{j}=\sqrt{\frac{{\sum_{i=1}^{m} (F_{ij}-\overline{F_{j}})}^{2}}{m}} j\in\left\{ 1,2,\ldots,n \right\}$  Here, $\sigma_{j}=\sqrt{\frac{{\sum_{i=1}^{m} (F_{ij}-\overline{F_{j}})}^{2}}{m}} j\in\left\{ 1,2,\ldots,n \right\}$  Here, is the arithmetic mean of the j. normalized objective values. Finally, the weight for each criterion is determined as follows. | $P_{i}=\sum_{j=1}^{n} (r_{ij})^{w_{j}}$  This Pi value also achieved according to the WASPAS multiplicative attitude. Calculate the relative weights of the alternatives using the aggregation strategies below. $k_{ia}=\frac{P_{i}+S_{i}}{\sum_{i=1}^{m} \left(P_{i}+S_{i}\right)}$  | The ideal and anti-ideal solutions are determined with following equations.  $v_{i}=maxs_{ij}$  $v_{ai}=mins_{ij}$  Deviations from ideal and anti-ideal solutions are computed by folowing Equations  $d^{+}={max v}_{i}-s_{ij}$  $d^{-}=s_{ij}-{min v}_{ai}$ |
| **4** | $c_{j}=\sigma_{j}\sum_{k=1}^{n} (1-\rho_{jk})$  $jw_{j}=\frac{c_{j}}{\sum_{k=1}^{n} c_{k}} j\in\left\{ 1,2,\ldots,n \right\}\in\left\{ 1,2,\ldots,n \right\}$ | $k_{ib}=\frac{S_{i}}{\min_{i} S_{i}}+\frac{P_{i}}{\min_{i} P_{i}}$ $k_{ic}=\frac{\lambda(S_{i})+(1-\lambda)(P_{i})}{(\lambda\max_{i} S_{i}+(1-\lambda)\max_{i} P_{i})}; 0\leq\lambda\leq1 .$   | The grades of the deviations for each alternative from anti-ideal and ideal solutions are computed as. $o_{i}^{+}=\sum_{j=1}^{n} d^{+}$  $o_{i}^{-}=\sum_{j=1}^{n} d^{-}$ The utility function for each alternative pertaining to the deviations from the optimal alternatives is computed as.  $K_{i}^{+}=\frac{o_{opt}^{+}}{o_{i}^{+}}$ $K_{i}^{-}=\frac{o_{opt}^{-}}{o_{i}^{-}}$ |
| **5** |  | The final ranking of the alternatives is determined based on ki, values (as more significant as better):   | The average deviation value for each alternative is computed as. The alternative with the highest $Q_{i}$ is determined as the best alternative.  $Q_{i}=\frac{K_{i}^{+}+K_{i}^{-}}{2}$ |
